# By invitation only – the case for breast cancer screening reminders for women over 69 years

**DOI:** 10.1186/1743-8462-5-23

**Published:** 2008-11-06

**Authors:** Carla Saunders, Monica Robotin, Sally Crossing

**Affiliations:** 1 The Cancer Council NSW, Woolloomooloo NSW, Australia; 2 School of Public Health, University of Sydney, Australia; 3 Cancer Voices NSW, Greenwich NSW, Australia

## Abstract

**Background:**

Breast cancer is the leading cause of cancer death in women in Australia. Early detection provides the best chance of reducing mortality and morbidity from the disease. Mammographic screening is a population health strategy for the early detection of breast cancer in Australia. Recruitment strategies such as regular advertising and biannual screening invitations are exclusively targeted at women aged 50 – 69 years. Even though they can participate, women 70 years or over are not invited or actively encouraged to undertake screening. Research has found that a routine letter of invitation increases the number of women participating in breast cancer screening.

**Methods:**

Cancer data analysis and a literature and policy review was conducted to assess age specific breast cancer mortality rates and the legitimacy of rationale used to limit invitations for breast cancer screening to women younger than 70 years.

**Results:**

The proportion of women over 69 years participating in the BreastScreen program is significantly less than rate of screening in the target age range (50–69 years). Evidence and data indicate that common justifications for limiting screening reminders to the target age range including life expectancy, comorbidities, effectiveness, treatment and cost are, for many women, unreasonable.

**Conclusion:**

There is now sufficient data to support a change in the targeted upper age range for breast cancer screening to improve the existing suboptimal surveillance in women aged over 69 years.

## Background

Breast cancer is the most common invasive cancer diagnosed in females in Australia and is the leading cause of cancer death in this group [1]. Age is the most important single risk factor for disease development and death rates increase consistently with advancing age [2]. In 2005, the death rate per 100,000 for women aged 40–44 years was 17.0 increasing to 66 per 100,000 in women aged 65–69 years and escalating to 182.0 deaths per 100,000 in women aged 85 years or over [2]. The annual number of years of potential life lost due to female breast cancer in Australia is over 32,000 [3]. Long life expectancy is regarded as an indicator of a successful society and an effective health care system [4].

Mammography has been demonstrated to reduce morbidity and mortality from breast cancer [5] through early disease detection, which, when managed effectively, leads to improved survival and less aggressive treatment [3]. At this time, mammography is the only accepted screening modality for breast cancer in average risk populations [6].

BreastScreen Australia is the national breast cancer screening program offering free mammography screening to asymptomatic women since 1991. A clear upper age limit has not been set for breast cancer screening in Australia; however, recruitment strategies are specifically targeted at women aged 50 – 69 years who are reminded to have a mammogram every two years via written invitations [7]. Women aged 40–49 and 70 years or over are not invited for screening, although they are able to participate [7]. Research has found that a routine letter of invitation increases the number of women participating in breast cancer screening [8,9].

## Methods

An examination of recent national cancer data was undertaken to assess age specific breast cancer mortality rates and a literature and policy review was conducted to assess the legitimacy of rationale used to limit invitations for breast cancer screening to women younger than 70 years of age.

## Results

**Figure 1:**
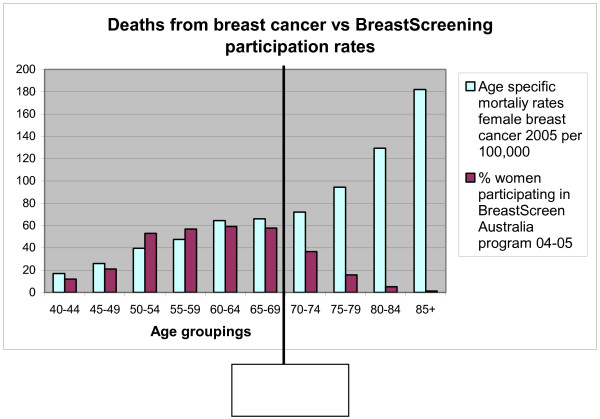
**A comparison of death rates from female breast cancer in 2005 with BreastScreen participation rates in 2004–5**. Data source: Ref [3] Australian Institute of Health and Welfare 2008. BreastScreen Australia monitoring report 2004–2005. Cancer series no. 42. Cat. no. CAN 37.

There has been a slow decline in the number of women older than 69 years participating in the national BreastScreen program over the past decade, with only 11% of women in this age group participating in 2004–5 [3]. This compares very poorly with participation rates of the targeted age range, which have steadily increased over the past decade and currently sit at almost 74% [3]. Figure 1 compares death rates from female breast cancer with BreastScreen participation rates, demonstrating that underscreening has a strong negative effect on breast cancer survival.

One reason given for targeting women 50–69 years for screening is that early detection in this age group offers women "a better chance of successful treatment and recovery" presumably, because of a higher likelihood of comorbid conditions, reduced treatment tolerance and shorter life expectancy in women above 69 years [10]. Another reason given is that most of the research showing mammographic screening effectiveness was conducted with women aged 50–69 years [10]. The national *BreastScreen *policy states that the age for screening will be "monitored and reviewed as new data become available" [5]. A program review is currently underway to assess the appropriateness of the existing *BreastScreen Australia *targeted age range [11].

### Effectiveness

In the past, elderly women have been significantly underrepresented in the evidence base used to determine the effectiveness of mammography. Researchers used age limits in recruitment as cut off points to minimise potential problems caused by comorbid health conditions and the risk of loss to follow up from unexpected early death [12]. This has contributed to the lack of clear policy guidelines and resultant under-surveillance of breast cancer in elderly women.

A recent study with over 860,000 participants aged between 70–75 years has shown that breast cancer screening using mammography reduces deaths from the disease in women in this age group. This study also found that screening did not cause significant harm by exposing the older women to over-diagnosis and over-treatment [13]. Numerous other investigations support targeted mammographic breast cancer screening in older age groups, [14-20] including a 2007 review [21] which found that there is now sufficient data to support regular screening above age 75 years based on individual assessment.

### Life expectancy

The size of the older population in Australia is growing, with females representing an increasing proportion of the population. At age 70, the average life expectancy of a woman living in Australia is over 15 years [22]. Many of these women, if in sound health, will live considerably longer than the average life expectancy and will remain at risk for breast cancer development and recurrence. Approximately 25% of women 75 years of age will live more than 17 years and 50% will live at least another 12 years [23]. There are approximately 56,000 female octogenarians currently living in Australia. This number is increasing and is predicted to reach approx 69,000 by 2020 and 107,000 by 2027 [23].

### Comorbidity

The existence of comorbid conditions has been used as rationale to exclude women over 69 years in breast cancer screening invitations. The findings of the most recent Australian Bureau of Statistics *National Health Survey *indicate that almost all people aged 65 years or older have at least one persistent health problem and around 80% reporting three or more [24]. The most frequently reported long term conditions are problems with eyesight, hayfever/allergic rhinitis, arthritis and back and disc disorders [24,25]. Considering the most commonly reported chronic conditions and the expected large heterogeneity in the health status of this older population, many women in older age groups will not have comorbidity severe enough to negate the benefits in survival and quality of life gained from breast cancer screening.

Research [26,27] and projections [28] based on trends of improvements in health generally, including changing levels of fitness in all age groups, indicate that the total burden of chronic disease will decline significantly in the next three to four decades. Moreover, gerontological research has now identified factors that can predict which women are likely to remain in good health and which may not. For example, progressive dependence on others for support in daily living activities can predict reduced survival over the intermediate term [29-31].

### Treatment

The ability of elderly women to endure cancer treatment has been raised in the breast screening debate. Yet, less invasive surgical procedures and better targeting of breast cancer treatments strengthen support for the involvement of older women in breast cancer screening programs [32,33]. A large body of evidence demonstrates that fit older women who have breast cancer can safely undergo the same treatments and show equal tolerance as their younger counterparts with comparable results. [34-40]

### Cost

Resource limitation has also been given as a possible underlying reason for not encouraging routine screening older women. However, cost-effectiveness analyses suggest that breast cancer screening after the age of 65 years reduces mortality at reasonable costs in women who are without significant comorbidities and that it is likely to be worthwhile until a woman has an estimated life expectancy of between five and 10 years [41,42]. Interventions to purposely foster participation in screening mammography among women 65 years and older are being researched internationally [43].

### Community views

In 1997, Glasziou highlighted the importance of involving target groups in screening decisions in order to develop "rational and acceptable policy" around breast cancer screening [44]. Breast Cancer Network Australia, a 22,000 member organisation, is the national organisation representing Australians affected by breast cancer. This group advocates for 'the availability of free mammography to be promoted to all women over 40, not just the target age of 50–69' [45]. Similarly, Cancer Voices NSW, the independent, peak advocacy organisation for all people affected by cancer in New South Wales, with over 4,000 members, advocates for an increase in breast cancer screening promotion by government. Moreover, this organisation seeks for 'the adoption by government of the principles of cancer consumer participation wherever decisions affecting them are made' [46]. The Breast Cancer Action Groups in NSW and Victoria have long advocated for screening reminders to be made available for women aged 70 and over [47].

The Australian community as a whole benefits from elderly members who are in good health and are given the opportunity to reach an average life expectancy at a minimum. Their contribution to childcare and other unpaid caring duties; financial, practical and emotional assistance to family members and friends, and voluntary work has been reported to add up to around 7% of the gross domestic product [48].

Overseas not-for-profit community support organisations also advocate for organised breast cancer screening for the elderly. The American Cancer Society advises women to continue breast cancer screening as long as they are in good health and are suitable candidates for cancer treatment [49] and the American Geriatrics Society [50,51] recommends that screening should be individualised, rather than age-driven alone, recommending no upper age limit for screening, as long as estimated life expectancy is 4 years or more.

## Discussion

Detection of early stage breast cancers offers the potential for longer survival and enhanced quality of life for many women irrespective of age. There is a pressing need to assemble all relevant evidence, including community needs, with regard to breast cancer screening in the elderly and translate it into a cohesive policy.

We acknowledge that at policy level, making decisions around screening age is a complex process, but age alone should not be a criterion for discontinuing invitations to attend regular breast screening. Screening may not be suitable for all, as some women may have other health problems that would reduce the long-term benefit of screening or may choose not to screen for other reasons. For others, screening may provide some additional life years, that might otherwise be lost. Regardless of which position women take, the opportunity to weigh up the potential benefits and risks of mammography needs to be available to all if they so choose.

Decision-making aids, in conjunction with opportunities to discuss these decisions with their healthcare providers can assist women define their individual position around whether they wish to attend repeat screening. This information can be fed back to BreastScreen through an opt-off process, closing the loop on consumer involvement and improving public communication. Continuing the recall system, while providing realistic opportunities for women to examine benefit-harm tradeoffs would ensure that breast cancer screening remains equitable in all age groups and that beneficiaries can avail themselves of this service, fully informed of the consequences of their decisions.

## Conclusion

With the growing number of older women in Australia, breast cancer will become an increasingly significant health concern, yet there is suboptimal surveillance of breast cancer in those aged over 69 years. Prompts to continue breast cancer screening are currently provided on the basis of chronological age alone. Continuing reminders after aged sixty nine and adopting a patient centred approach (which takes the form of an informed, shared, decision-making process between a woman and her medical practitioner) for individual decisions about screening is proposed.

## Competing interests

The authors declare that they have no competing interests.

## Authors' contributions

CS undertook the data analysis and literature and policy review, and was the main author of this paper; MR supervised the analysis and review and contributed to the development, drafting and editing of the paper; SC provided input and important comments on drafts of the manuscript.
